# Slug increases sensitivity to tubulin-binding agents via the downregulation of βIII and βIVa-tubulin in lung cancer cells

**DOI:** 10.1002/cam4.68

**Published:** 2013-03-01

**Authors:** Daisuke Tamura, Tokuzo Arao, Tomoyuki Nagai, Hiroyasu Kaneda, Keiichi Aomatsu, Yoshihiko Fujita, Kazuko Matsumoto, Marco A De Velasco, Hiroaki Kato, Hidetoshi Hayashi, Shuhei Yoshida, Hideharu Kimura, Yoshimasa Maniwa, Wataru Nishio, Yasuhiro Sakai, Chiho Ohbayashi, Yoshikazu Kotani, Yoshihiro Nishimura, Kazuto Nishio

**Affiliations:** 1 Department of Genome Biology, Kinki University Faculty of MedicineOsaka, Japan; 2 Division of Respiratory Medicine, Department of Internal Medicine, Kobe University Graduate School of MedicineKobe, Japan; 3 Division of Thoracic Surgery, Kobe University Graduate School of MedicineKobe, Japan; 4 Division of Pathological Oncology, Kobe University Graduate School of MedicineKobe, Japan

**Keywords:** Anoikis, lung cancer, slug, tubulin-binding agents, tumor growth

## Abstract

Transcription factor *Slug*/*SNAI2* (*snail homolog 2)* plays a key role in the induction of the epithelial mesenchymal transition in cancer cells; however, whether the overexpression of *Slug* mediates the malignant phenotype and alters drug sensitivity in lung cancer cells remains largely unclear. We investigated *Slug* focusing on its biological function and involvement in drug sensitivity in lung cancer cells. Stable *Slug* transfectants showed typical morphological changes compared with control cells. *Slug* overexpression did not change the cellular proliferations; however, migration activity and anchorage-independent growth activity with an antiapoptotic effect were increased. Interestingly, stable *Slug* overexpression increased drug sensitivity to tubulin-binding agents including vinorelbine, vincristine, and paclitaxel (5.8- to 8.9-fold increase) in several lung cancer cell lines but did not increase sensitivity to agents other than tubulin-binding agents. Real-time RT-PCR (polymerase chain reaction) and western blotting revealed that *Slug* overexpression downregulated the expression of βIII and βIVa-tubulin, which is considered to be a major factor determining sensitivity to tubulin-binding agents. A luciferase reporter assay confirmed that *Slug* suppressed the promoter activity of *βIVa-tubulin* at a transcriptional level. *Slug* overexpression enhanced tumor growth, whereas *Slug* overexpression increased drug sensitivity to vinorelbine with the downregulation of βIII and βIV-tubulin in vivo. Immunohistochemistry of Slug with clinical lung cancer samples showed that *Slug* overexpression tended to be involved in response to tubulin-binding agents. In conclusion, our data indicate that *Slug* mediates an aggressive phenotype including enhanced migration activity, anoikis suppression, and tumor growth, but increases sensitivity to tubulin-binding agents via the downregulation of βIII and βIVa-tubulin in lung cancer cells.

## Introduction

The epithelial-mesenchymal transition (EMT), a developmental process in which epithelial cells reduce intercellular adhesions and acquire fibroblastic properties, has emerged to play important roles in the development of the invasive and metastatic potentials of cancer progression 1–3. During the EMT process, epithelial cells lose the expression of E-cadherin and other components of epithelial cell junctions, adopt a mesenchymal cell phenotype, and acquire motility as well as invasive properties 4. The snail family of zing finger transcription factors, *Snail/SNAI1* (*snail homolog 1*) and *Slug/SNAI2* (*snail homolog 2*), are highly conserved transcription repressors implicated in embryonic development and tumorigenesis, and they are considered to be key EMT-inducible transcription factors 5.

Many clinicopathological studies have demonstrated positive correlations between the expressions of *Slug* and poor clinical outcomes in breast, ovary, colorectal cancer, and melanoma 1. In lung cancer, the elevated expression of *Slug* messenger ribonucleic acid (mRNA) in cancer tissue was significantly associated with postoperative relapse and a shorter patient survival period 6. These accumulating data strongly suggest that *Slug* is a poor prognostic factor in many solid cancers and may be involved in the process of metastasis. Meanwhile, the EMT is thought to be involved in drug sensitivity to several anticancer agents 7. Regarding sensitivity to gemcitabine, mesenchymal-type cancer cells are reportedly associated with gemcitabine resistance in pancreatic cancer cells 8. A recent study showed that patterns of sensitivity and resistance to three conventional chemotherapeutic agents (gemcitabine, 5-fluorouracil, and cisplatin) in pancreatic cancer cell lines were closely associated with the EMT phenotype 9. The mechanism of resistance to gemcitabine has been shown to involve the activation of notch signaling, which is mechanistically linked with the mesenchymal-chemoresistance phenotype of pancreatic cancer cells 10. Another association with the EMT has been found in epidermal growth factor receptor (EGFR)-targeting drugs for the treatment of lung cancer. A clinical trial has revealed that lung cancer cells with strong E-cadherin expression exhibit a significantly longer time to progression after EGFR-tyrosine kinase inhibitor (EGFR-TKI) treatment 9. Other studies on EGFR-targeting drugs have demonstrated that mesenchymal-type lung cancer cells exhibit an EMT-dependent acquisition of platelet-derived growth factor, fibroblast growth factor receptor, and transforming growth factor beta receptor signaling pathways 11, and integrin-linked kinase is a novel target for overcoming hepatocellular carcinoma resistance to EGFR inhibition 12. Thus, baseline cellular characteristics based on the EMT phenotype might be useful not only as prognostic biomarkers for a malignant phenotype, but also for predictive markers of sensitivity to anticancer agents.

Collectively, *Slug* overexpression likely mediates a malignant phenotype and is related to drug sensitivity in several solid cancers; however, detailed studies on the roles of *Slug* in lung cancer cells remain largely unclear. In this study, we investigated the biological effects of *Slug* on the cellular phenotype and drug sensitivity in lung cancer cells.

## Materials and Methods

### Reagents

Vinorelbine (VNR), vincristine (VCR), paclitaxel (PTX), and cisplatin were purchased from Wako Pure Chemical Industries (Osaka, Japan). AG1478, an EGFR-TKI, was purchased from Biomol International (Plymouth Meeting, PA). Each chemical agent was dissolved in dimethylsulfoxide (DMSO) for use in the in vitro experiments, and VNR was dissolved in phosphate-buffered saline (PBS) for use in the in vivo experiment.

### Cell lines and cultures

The NCI-H1299 (H1299) and A549 cell lines were obtained from the American Type Culture Collection (Manassas, VA). Ma-1 cells were kindly provided by Dr. E. Shimizu (Tottori University, Yonago, Japan). A549, Ma1, and H1299 were maintained in RPMI 1640 (Sigma, St. Louis, MO) with 10% heat-inactivated fetal bovine serum (FBS) and 0.1% gentamicin–amphotericin B. All the cell lines were incubated at 37°C with humidified 5% CO_2_.

### Paraffin sections

All primary non-small cell lung cancer specimens were derived from patients who underwent surgery at Kobe University Hospital between April 2001 and December 2005. We examined 10 samples of patients who took chemotherapy including TBAs before or after surgery. Baseline characteristics of the samples are presented in Table S1. The institutional review board approval was obtained for this study.

### Plasmid construction, viral production, and stable transfectants

The cDNA fragment encoding human full-length Slug was isolated from human corneal epithelial cells using polymerase chain reaction (PCR) and Prime STAR HS DNA polymerase (TaKaRa, Otsu, Japan) with 5′-GG GAA TTC GGC GCC ATG CCG CGC TCC TTC CTG GTC-3′ and 5′-CC CTC GAG CGT CAC TCA GTG TGC TAC ACA GCA GCC-3′ as sense and antisense primers, respectively. The methods used in this section have been previously described 13. In brief, the sequences of the PCR-amplified DNAs were confirmed by sequencing after cloning into a pCR-Blunt II-TOPO cloning vector (Invitrogen, Carlsbad, CA). Slug cDNA was cut out and transferred into a pQCLIN retroviral vector (BD Biosciences Clontech, San Diego, CA) together with an enhanced green fluorescent protein (EGFP) following internal ribosome entry site sequence (IRES) to monitor the expression of the inserts indirectly. A pVSV-G vector (Clontech, Palo Alto, CA) for the constitution of the viral envelope and the pQCXIX constructs were cotransfected into the GP2-293 cells using the FuGENE6 transfection reagent (Roche Diagnostics, Basel, Switzerland). The stable transfectants expressing EGFP only or EGFP and Slug in each cell line were designated as A549/EGFP, A549/Slug, Ma1/EGFP, Ma1/Slug, H1299/EGFP, and H1299/Slug. These stable cell lines were established from bulk transfected cells, and not from cloned single cells.

### Real-time RT-PCR

The methods used in this section have been previously described 13. The primers used for real-time RT-PCR were purchased from TaKaRa (Table S2). The experiment was performed in triplicate.

### Western blotting

The antibodies used for western blotting were as follows: anti-Slug, anti-E-cadherin, anti-N-cadherin, anti-vimentin, anti-fibronectin, anti-β-actin, anticleaved or noncleaved caspase3, anticleaved or noncleaved poly (ADP-ribose) polymerase (PARP), anti-β-tubulin, and anti-βIII-tubulin (Cell Signaling, Beverly, MA), and anti-βI, II, IV-tubulin (Sigma). The western blot analysis was performed as described previously 14. The experiment was performed in duplicate.

### Immunofluorescence staining

The methods used in this section have been previously described 15. Cells were treated with DAPI (6-diamidino-2-phenylindole) to stain the nucleus and photographed using fluorescent microscopy (IX71; Olympus, Tokyo, Japan). The experiment was performed in triplicate.

### In vitro growth inhibition assay and migration assay

Growth inhibition was evaluated using an MTT assay, as described previously 16. The methods used in migration assay have been previously described 13. The experiment was performed in triplicate.

### Anchorage-independent growth assay

Anchorage-independent cellular growth was evaluated using an ultra-low attachment surface 6-well plate (Corning® Inc., Lowell, MA). In brief, cells were seeded onto the plates containing RPMI 1640 with 10% FBS. After incubation for 1 week at 37°C, the cells were stained with trypan blue, then observed immediately using a light microscope. The experiment was performed in triplicate. To show numerical comparisons for viable cells, we also performed an MTT assay.

### Luciferase reporter assay

The method used in this section has been previously described 17. *TUBB4* promoter fragments (2.5 kbp) were isolated using PCR with the following primers: sense, 5′-GGG GTA CCG TTG AGA GCC ACT GGT CAA ATT TAG TG-3′ and antisense, 5′-GGA AGC TTG CGG CGA GGG TGG AAG ATG CGG CGG AG-3′ for *TUBB4*. The *TUBB4* promoter fragments were cut between the KpnI and HindIII in *TUBB4* promoter restriction sites and were transferred into the luciferase reporter vector pGL4.14 (Promega, Madison, WI). All the sequences were verified using DNA sequencing. The empty and *TUBB4* promoter-containing reporter vectors were designated as pGL4.14-mock and pGL4.14-TUBB4, respectively. All the samples were examined in triplicate.

### Chromatin immunoprecipitation

Chromatin immunoprecipitation (ChIP) was carried out using the ChIP-IT™ Express Enzymatic Kit (Active Motif, Carlsbad, CA) according to the manufacturer's protocol. A549/EGFP and A549/Slug cells were used for analysis. Immunoprecipitation was done using an anti-Slug antibody (Santa Cruz Biotechnology, Santa Cruz, CA) and a control IgG antibody (Cell Signaling). Two putative regions (E2-box #1, −1289 to −1157 and E2-box #2, −683 to −547) in the *TUBB4* promoter-containing E2-box sequences were amplified with the following respective primers: E2-box #1 (forward) 5′-GCC TGG ACA ACA TAG CGA GAC CCC ATC TC-3′ and (reverse) 5′-CCT CAA TGT CCT GGG CTC AAG CAA TCC TC-3′, E2-box #2 (forward) 5′-TCC CAA TAT GAA CCC AGC TGT CTT ACT CCT C-3′ and (reverse) 5′-CCC ATG ATT GGG TGC AGA TGC TGG TGG GCT-3′. As a control, the *GAPDH* 2nd intron promoter was amplified with the following primers: (forward) 5′-AAT GAA TGG GCA GCC GTT AG-3′ and (reverse) 5′-AGC TAG CCT CGC TCC ACCTGA C-3′.

### Xenograft studies

Nude mice (BALB/c nu/nu; 6-week-old females; CLEA Japan Inc., Tokyo) were used for the in vivo studies and were cared for in accordance with the recommendations for the handling of laboratory animals for biomedical research compiled by the Committee on Safety and Ethical Handling Regulations for Laboratory Animal Experiments, Kinki University. Mice were subcutaneously inoculated with a total of 4 × 10^6^ A549/EGFP (*n =* 10) or A549/Slug cells (*n =* 10). Two weeks after inoculation, the mice were randomized according to tumor size into two groups to equalize the mean pretreatment tumor size between the two groups (vehicle control or VNR treatment, *n =* 5 in each group). The mice were then treated with VNR (5 mg/kg/week, i.p.) or the vehicle control (PBS, i.p.) once a week for 3 weeks (administered on days 1, 8, and 15). On day 60, the mice transplanted with A549/EGFP cells were euthanized and the tumor specimens were collected for immunohistochemistry. The mice transplanted with A549/Slug cells were euthanized on day 25 because of ethical considerations, as the tumors grew much faster in these animals. The tumor volume was calculated as the length × width^2^ × 0.5 and was assessed every 2–3 days.

### Immunohistochemistry analysis

Paraffin sections were immunohistochemical stained with rabbit anti-Slug (1:100, Cell Signaling) using standard methods 18. Signal detection was achieved using the avidin–biotin peroxidase complex assay (ABC Elite Kit, Vector Laboratories, Burlingame, CA) according to the manufacturer's instructions. Slides were then developed with diaminobenzidine (DAB, Invitrogen, Carlsbad, CA) and counterstained with 10% hematoxylin. Immunohistochemically stained sections were examined microscopically and random fields of tumor tissue were identified, digitally captured, and saved for quantification using ImageJ image analysis software (http://rsb.info.nih.gov/ij/). Separate images consisting of positive or background signals (corresponding to DAB and hematoxylin, respectively) were generated from RBG color space original images using a color deconvolution plugin 19. Suitable thresholds were adjusted and kept constant for each slide, and binary masks were created. The image analysis (IA) score representing the area of pixels corresponding to DAB (positive cells)/area of pixels corresponding to hematoxylin (negative cells) was calculated and averaged for each individual case.

### Statistical analysis

The statistical analyses were performed using Microsoft Excel (Microsoft) to calculate the SD and to test for statistically significant differences between the samples using a Student *t*-test. A *P* value of <0.05 was considered statistically significant.

## Results

### Slug mediated the EMT in A549 cells

**Figure 1:**
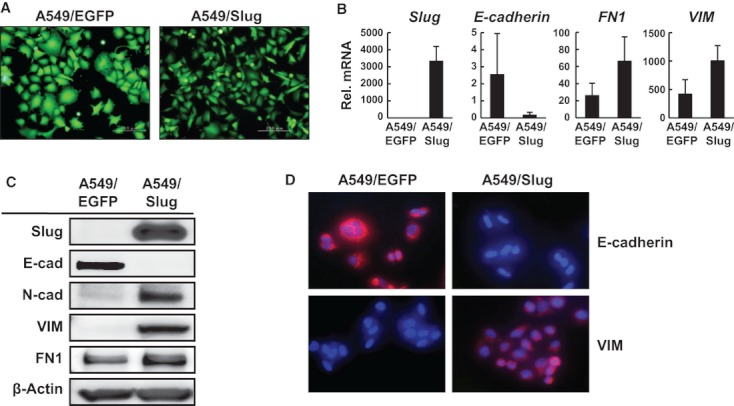
Slug mediates the epithelial mesenchymal transition (EMT) in A549 cells. Stable transfectants expressing enhanced green fluorescent protein (EGFP) only or EGFP and Slug were designated as A549/EGFP and A549/Slug, respectively. (A) Morphological changes of A549/EGFP and A549/Slug cells. (B) The mRNA expression levels of *Slug*, *E-cadherin*, *fibronectin 1,* and *vimentin* were determined using real-time RT-PCR in both cell lines. *GAPD* was used to normalize the expression levels. *E-cad*: *E-cadherin*, *FN1*: *Fibronectin 1*, *VIM*: *Vimentin*, Rel mRNA: normalized mRNA expression levels (*target genes*/*GAPD* × 10^3^). The data shown represents the average ± SD of three independent experiments. (C) Western blot analysis for Slug, E-cadherin, N-cadherin, vimentin, and fibronectin. β-actin was used as an internal control. (D) Immunofluorescence staining of E-cadherin (red) and vimentin (red). Cells were treated with DAPI (blue, 6-diamidino-2-phenylindole) to stain the nucleus. PCR, polymerase chain reaction; SD, standard deviation; mRNA, messenger ribonucleic acid.

To investigate the potential roles of Slug in lung cancer cells, we retrovirally introduced the *Slug* gene into A549 cells. The stable transfectants expressing EGFP only or EGFP and Slug and were designated as A549/EGFP and A549/Slug, respectively. Slug overexpression induced EMT-like changes in cell morphology including the elongation of the cell shape and cell scattering, which are characteristic features of cells undergoing EMT (Fig. 1A). Real-time RT-PCR demonstrated that Slug overexpression markedly downregulated the mRNA levels of *E-cadherin*, whereas the mesenchymal markers *fibronectin 1* and *vimentin* were upregulated, compared with A549/EGFP (Fig. 1B). Western blotting showed that Slug overexpression mediated the downregulation of E-cadherin and the upregulation of N-cadherin, vimentin, and fibronectin (Fig. 1C). Immunofluorescence staining also showed the downregulation of E-cadherin and the upregulation of vimentin (Fig. 1D). These results indicated that Slug induces EMT in A549 cells.

### Slug enhanced anchorage-independent growth and cellular migration

**Figure 2:**
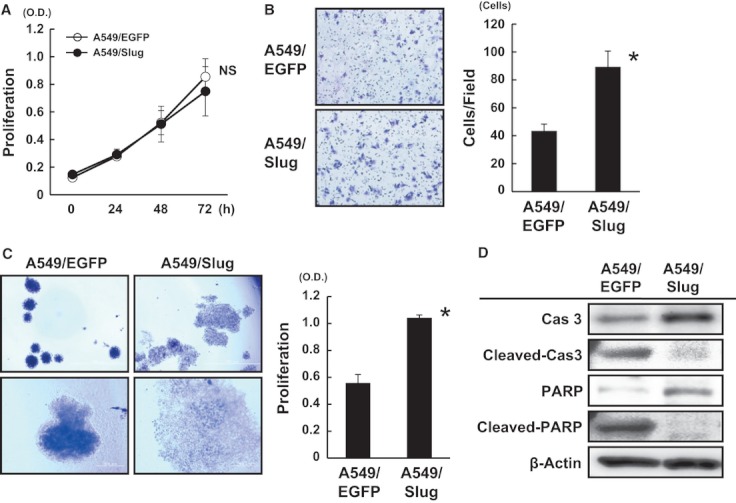
Slug enhanced anchorage-independent growth and cellular migration, but not cellular proliferation. (A) Cellular proliferation was examined using an MTT assay. No significant difference was observed between A549/EGFP and A549/Slug cells. The data shown represent the average ± SD of three independent experiments. ns: not significant. (B) A migration assay was performed using the Boyden-chamber method and both cell lines. The left panels show representative data. The data shown represent the average ± SD of three independent experiments. *P < 0.05. (C) Anchorage-independent cell growth was evaluated using an ultra-low attachment surface plate. The cells were seeded onto a plate containing RPMI 1640 with 10% FBS and cultured for a week. The cells were stained with trypan blue and observed immediately using a light microscope. The upper panel shows pictures obtained at a low-power field, and the lower panel shows pictures obtained at a high-power field. The cellular proliferation was evaluated using an MTT assay (right panel). The data show the average ± SD of three independent experiments. *P < 0.05. (D) Western blot analysis for caspase 3 and PARP with cleaved or noncleaved forms. The cells were cultured on low attachment surface plates under anchorage-independent growth conditions for 24 h and collected for analysis. Cas 3, caspase 3. β-actin was used as an internal control. EGFP, enhanced green fluorescent protein; SD, standard deviation; FBS, fetal bovine serum; PARP, poly (ADP-ribose) polymerase.

We examined the cellular growth, migration activity, anchorage-independent cell growth, and the effect on apoptosis using A549/EGFP and A549/Slug cells. No difference was seen in cellular growth between these cells, indicating that Slug does not enhance cellular growth in vitro (Fig. 2A). Cellular migration activity of the A549/Slug cells was significantly enhanced, compared with the control cells (Fig. 2B). As anchorage-independent growth is considered a hallmark of the EMT, we evaluated the effect of Slug overexpression. Control cells formed dense spheroid-like clusters under anchorage-independent conditions, whereas A549/Slug cells exhibited notably different loose clusters of cells (Fig. 2C). Trypan blue staining revealed that A549/EGFP cells included many stained dead cells, but A549/Slug cells did not; these results were confirmed by an MTT assay (Fig. 2C). Western blotting showed that Slug overexpression decreased the protein expression of cleaved caspase and cleaved PARP under anchorage-independent conditions, suggesting that Slug suppressed anoikis (Fig. 2D). These results indicated that Slug increased the migration activity and anchorage-independent growth activity with antiapoptotic effects in lung cancer cells.

### Slug sensitized lung cancer cells to tubulin-binding agents

**Figure 3:**
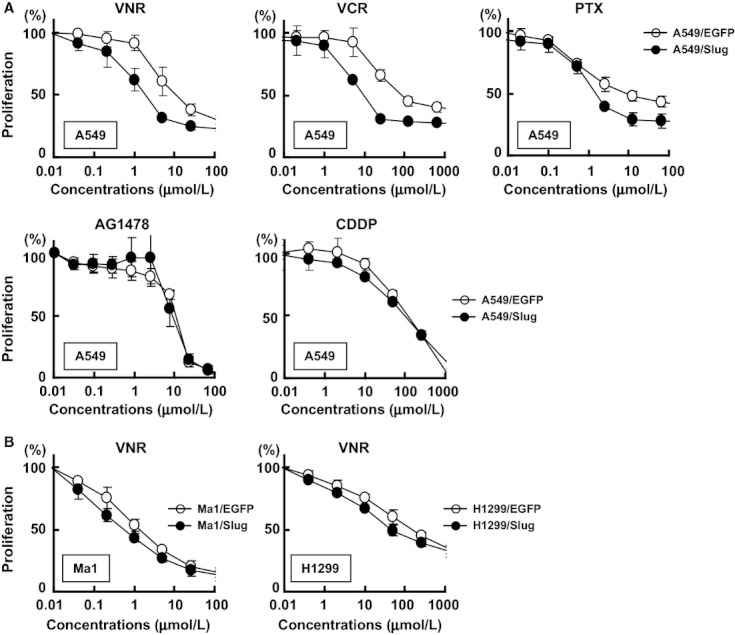
Slug sensitized lung cancer cells to tubulin-binding agents (TBAs) including vinorelbine, vincristine, and paclitaxel. (A) Growth inhibition by anticancer agents (vinorelbine, vincristine, paclitaxel, AG1478, and cisplatin) was evaluated using an MTT assay against A549/EGFP and A549/Slug cells. The data shown represent the average ± SD of three independent experiments. (B) Growth inhibition by vinorelbine was also confirmed in other stable transfectants: Ma1/EGFP, Ma1/Slug, H1299/EGFP, and H1299/Slug cells. The data shown represent the average ± SD of three independent experiments. VNR, vinorelbine; VCR, vincristine; PTX, paclitaxel. AG1478: an EGFR tyrosine kinase inhibitor, CDDP, cisplatin; EGFP, enhanced green fluorescent protein; SD, standard deviation.

Accumulating data indicate that the baseline mesenchymal phenotype is closely associated with resistance to chemotherapy; however, little is known about how Slug overexpression itself induces changes in drug sensitivity. Therefore, we evaluated the sensitivity of A549/EGFP and A549/Slug cells to VNR, VCR, PTX, cisplatin, and the EGFR-TKI. No significant differences were observed in the IC_50_ values of cisplatin and the EGFR-TKI to A549 cells (Fig. 3A). Unexpectedly, Slug overexpression sensitized the cells to VNR, VCR, and PTX by about 10-fold (Fig. 3A). To confirm whether Slug sensitizes other lung cancer cell lines to tubulin-binding agents (TBAs), we established another line of stable transfectants expressing EGFP only or EGFP and Slug using Ma1 and H1299 lung cancer cell lines (Ma1/EGFP, Ma1/Slug, H1299/EGFP, and H1299/Slug) as well as A549 cells and examined the drug sensitivity. Although the differences were relatively small compared with those in A549 cells, Slug overexpression sensitized both Ma1 and H1299 cells to TBAs (two- to fourfold of IC_50_, Fig. 3B). The IC_50_ values (μmol/L) in A549/GFP were as follows: VNR, 11.1 ± 5.5; VCR, 86.7 ± 29.1; PTX, 9.2 ± 1.9; AG1478, 11.1 ± 0.9; and CDDP, 127.2 ± 17.3. The IC_50_ values in A549/Slug were as follows: VNR, 1.9 ± 0.6; VCR, 9.7 ± 3.1; PTX, 1.5 ± 0.2; AG1478, 9.2 ± 2.8; and CDDP, 104.3 ± 13.0. The IC_50_ values for VNR were 1.4 ± 0.4 in Ma1/GFP, 0.6 ± 0.2 in Ma1/Slug, 15.0 ± 4.3 in H1299/GFP, and 4.8 ± 3.8 in H1299/Slug.

### Slug downregulated the expression levels of βIII and βIV-tubulin

**Figure 4:**
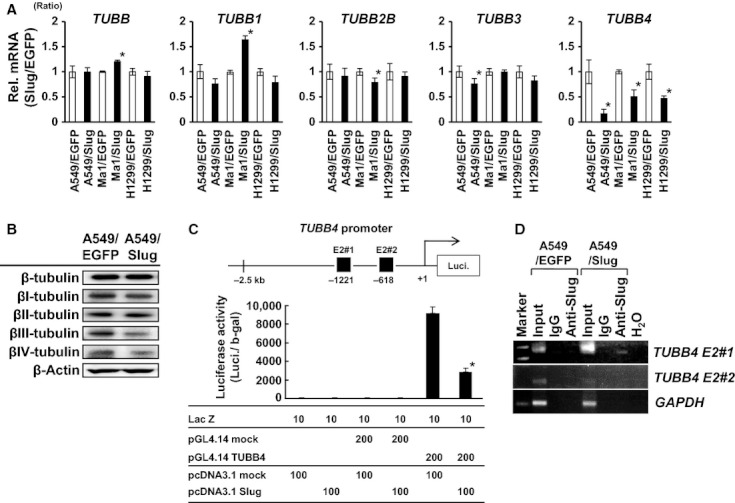
Slug downregulates the expression of βIII-tubulin/TUBB3 and βIVa-tubulin/TUBB4. (A) The mRNA expression levels of *TUBB*, *TUBB1*, *TUBB2B, TUBB3,* and *TUBB4* were determined using real-time RT-PCR in A549/EGFP, A549/Slug, Ma1/EGFP, Ma1/Slug, H1299/EGFP, and H1299/Slug cells. *GAPD* was used to normalize the expression levels. Rel mRNA: normalized mRNA expression levels (*target genes*/*GAPD* × 10^3^). The data shown represent the ratio of mRNA expression in Slug-overexpressing cells/EGFP cells with average ± SD of three independent experiments. *TUBB*, βI-tubulin isotypes; *TUBB1*, βVI-tubulin; *TUBB2B*, βII-tubulin; *TUBB3*, βIII-tubulin; *TUBB4,* βIVa-tubulin. (B) Western blot analysis for β-tubulin isotypes. βIII-tubulin and βIV-tubulin expression were suppressed in A549/Slug cells. β-actin was used as an internal control. (C) The *TUBB4* promoter activity was examined using a luciferase reporter assay. Luciferase vectors with either an empty or a *TUBB4* promoter (pGL4.14-mock or pGL4.14-TUBB4) that includes two E2-box sequences (E2-box #1 and E2-box #2) were transiently cotransfected with a mock or *Slug* expression plasmid (pcDNA3.1-mock or pcDNA3.1-Slug) expressing β-galactosidase as an internal control. The results were normalized to β-galactosidase activity and are representative of at least three independent experiments. *P < 0.05. (D) Chromatin immunoprecipitation (ChIP) of Slug on the promoter of *TUBB4*. A549/EGFP and A549/Slug cells were used for analysis. Two putative regions in the *TUBB4* promoter that contain the E2-box sequences (E2#1 and E2#2) were amplified using PCR. Primers to the *GAPDH* promoter were used as the control. Inputs, 1% of chromatin used for ChIP; H_2_O, no DNA templates. EGFP, enhanced green fluorescent protein; SD, standard deviation; PCR, polymerase chain reaction; mRNA, messenger ribonucleic acid.

Several studies have shown that the decreased expression of βIII and βIV-tubulin in cancer cells is involved in increased sensitivity to TBAs, and this β-tubulin expression is considered to be the major factor determining sensitivity to TBAs 20–24. Therefore, we hypothesized that Slug modulates the expression levels of the β-tubulin isotype (symbol, gene name, protein name, and accession number are shown in Table S3), thereby increasing the sensitivity to TBAs. Real-time RT-PCR revealed that Slug overexpression did not change the mRNA expressions of *TUBB*, *TUBB1,* and *TUBB2B*, while Slug significantly downregulated the expressions of *TUBB3* and *TUBB4* in A549/Slug, compared with in A549/EGFP cells (Fig. 4A). The mRNA expression of *TUBB*, *TUBB1,* and *TUBB2B* was changed in Ma1/Slug cells, but not changed in H1299/Slug cells. *TUBB4* expression was downregulated in all three cell lines (Fig. 4A). Western blotting also demonstrated that the protein expressions of β-tubulin, βI-tubulin, and βII-tubulin were not modulated by Slug, but βIII and βIV-tubulin were downregulated in A549/Slug cells (Fig. 4B).

### Slug suppresses the promoter activities of *TUBB4*

To address the question of whether Slug directly suppresses the transcription activities of *TUBB4*, a luciferase reporter assay was performed. The *TUBB4* promoter activity was suppressed by about 30%, when cotransfected with a Slug expression vector, compared with an empty vector (Fig. 4C). The result indicates that Slug directly suppresses the transcriptional activities of *TUBB4* at transcriptional levels. Two E2-box sequences (CAGGTG/CACCTG) are known as the classical binding site of Slug 25. The *TUBB4* promoter region contains two E2-box sequences at −1221 (E2-box #1) and −618 (E2-box #2) (Fig. 4C). To determine whether Slug directly binds to *TUBB4* promoter, we performed the ChIP assay against the two putative E2-box regions. The ChIP assay revealed that Slug was bound for the E2-box1 region and the interaction could not be detected for E2-box2 region (Fig. 4D). The result indicates that Slug binds to the *TUBB4* promoter and upregulates *TUBB4* transcriptional activity. Regarding the *TUBB3*, one E-box motif “CAGGTG” was found in the proximal *TUBB3* promoter at −643. This suggests that a similar mechanism of transcriptional regulation may exist for *TUBB3* transcriptional activation as well as *TUBB4*, although a detailed analysis was not performed. Further study on *TUBB3* promoter is warranted.

### Slug enhanced tumor growth but sensitized cells to vinorelbine in vivo

**Figure 5:**
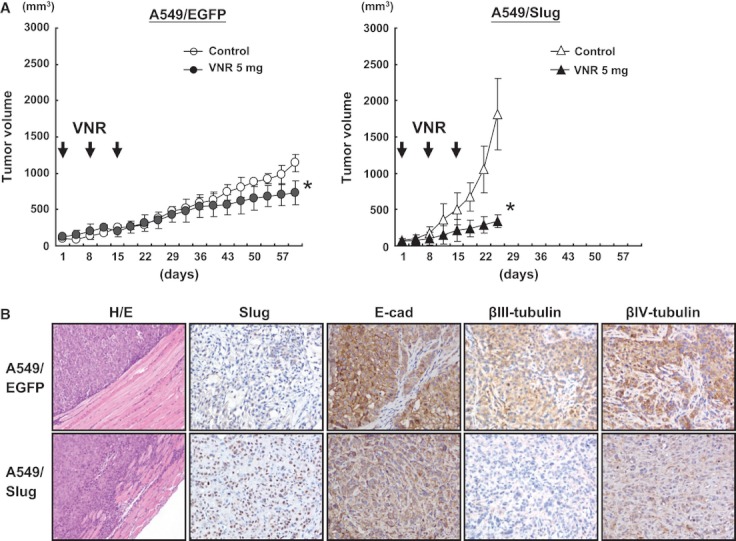
Slug enhanced tumor growth but sensitized cells to vinorelbine in vivo. (A) Tumor volumes of A549/EGFP or A549/Slug tumors treated with the vehicle control or vinorelbine. A total of 4 × 10^6^ A549/EGFP or A549/Slug cells were subcutaneously inoculated into the right flank of each mouse (A549/EGFP, *n =* 10; A549/Slug, *n =* 10). Two weeks after inoculation, the mice were then treated with vinorelbine (*n =* 5, 5 mg/kg/week, i.p.) or the vehicle control (*n =* 5, PBS, i.p.) once a week for 3 weeks. The tumor volume was calculated as the length × width^2^ × 0.5 and was assessed every 2–3 days. *P < 0.05. (B) HE, Slug, E-cadherin, βIII-tubulin, and βIV-tubulin staining of tumor specimens inoculated with A549/EGFP or A549/Slug cells. E-cad: E-cadherin. Original images were captured at ×200 magnification except for HE staining which was captured at ×40 magnification. EGFP, enhanced green fluorescent protein; PBS, phosphate-buffered saline.

We evaluated the Slug-mediated aggressive phenotype and increased drug sensitivity to VNR in vivo. Slug overexpression markedly enhanced the tumor growth in vivo (A549/EGFP, 378 ± 37 mm^3^ and A549/Slug, 1820 ± 491 mm^3^), supporting the idea that Slug induces an aggressive phenotype (Fig. 5A). On the other hand, VNR treatment weakly inhibited the tumor growth in A549/EGFP tumors, compared with in the vehicle control (19% of tumor reduction), while VNR treatment markedly inhibited tumor growth in A549/Slug tumors (64% of tumor reduction), clearly indicating that Slug overexpression markedly increased the drug sensitivity to VNR in vivo, similar to the results observed in vitro.

Immunohistochemical staining confirmed the forced expression of Slug and the downregulation of E-cadherin in A549/Slug tumors (Fig. 5B). In addition, the marked downregulation of βIII and βIV-tubulin was observed in A549/Slug tumors, compared with in A549/EGFP tumors, similar to the results observed in vitro (Fig. 5B). Collectively, these results indicate that Slug mediates an aggressive phenotype but increases sensitivity to TBAs via the transcriptional downregulation of βIII and βIV-tubulin in lung cancer cells.

### Slug expression tended to be involved in response to tubulin-binding agents in clinical lung cancer samples

**Figure 6:**
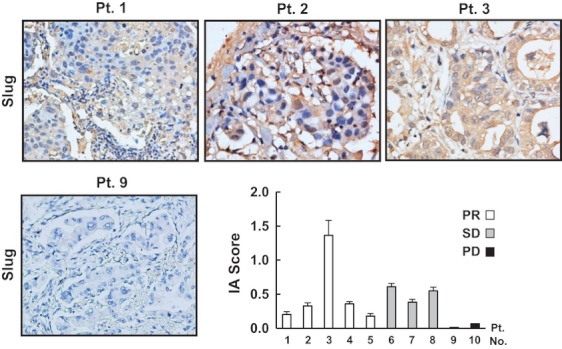
Slug expression in clinical samples of lung cancer. Lung cancer tissues were stained with the anti-Slug antibody using immunohistochemistry. Diffuse expression of Slug was observed in the specimens of Patients 1, 2, and 3, whereas Slug was not expressed in the lung tissue of Patient 9. Expression levels of Slug were quantified and shown as graph according to the response to tubulin-binding agents. IA Score, the image analysis score; PR, partial response; SD, stable disease; PD, progressive disease. Original images were captured at ×400 magnification.

We examined 10 clinical lung cancer samples from patients who undertook chemotherapy including TBAs. Immunohistochemistry of Slug showed that the lowest two patients in Slug staining had response of progressive disease, while other patients whose samples had higher expression of Slug resulted in partial response or stable disease against TBAs (Fig. 6). This result suggested that Slug expression is likely to be involved in drug response to TBAs in clinical samples.

## Discussion

The EMT has attracted the attention of researchers because of its involvement in the importance of drug sensitivity to anticancer agents in oncology. Several studies have shown that the EMT may be a determinant of the sensitivity of cancers to several anticancer agents such as EGFR-TKI, 5-flurouracil (5-FU), gemcitabine, and cisplatin 8,10,11,26–28, indicating that cancer cells with acquired resistance to anticancer agents have likely transitioned to a mesenchymal phenotype. However, little is known about the sensitivity of cells to TBAs. Unexpectedly, Slug overexpression did not mediate drug resistance but clearly sensitized lung cancer cells to TBAs in our investigation. Generally, several mechanisms have been suggested to be involved in drug sensitivity or resistance to TBAs, and some tubulin isotypes have been reported to be relevant to drug sensitivity to TBAs 20–24,29. To date, at least nine α-tubulin and seven β-tubulin isotypes have been described, with a complex pattern of distribution in various tissues. The β-tubulin isotypes differ from each other mainly according to differences in amino acid sequences clustered within the last 15 C-terminal residues, which constitute the isotype-defining domain. Some β-tubulin isotypes are widely expressed in many tissues (*βI*, *IVb,* and maybe *V*), while other isotypes are restricted to specific tissues (*βII*, *III*, and *IVa*, neuronal; *βIV*, hemopoietic 30). Among these isotypes of tubulin, the most intensively investigated mechanism of drug sensitivity to TBAs is the expression level of βIII and βIV-tubulin 22–24,31. We found that Slug increased the sensitivity to TBAs by about 10-fold in vitro. In addition, an increased sensitivity to VNR and the downregulation of βIII and βIV-tubulin in tumor specimens were observed in vivo. Our finding suggests that the suppression of βIII and βIVa-tubulin by Slug overexpression may contribute to the increased sensitivity to TBAs. ABC transporters are now considered to be involved in drug resistance to anticancer agents including TBAs, therefore we examined the mRNA expression levels of *ABCB1*, *ABCG2*, *ABCA1*, *ABCA2,* and *ABCC1* on A549/EGFP and A549/Slug cells using real-time RT-PCR. The mRNA expression of *ABCA1* was upregulated whereas expression levels of *ABCG2* and *ABCC1* were downregulated in A549/Slug cells, while the expression levels of *ABCB1* and *ABCA2* were very low (data not shown). The regulation of ABC transporters by Slug needs further study.

Anoikis suppression, survival from cell death induced by inappropriate or loss of cell adhesion, is likely to be a prerequisite for cancer cells to successfully metastasize to distant sites 32. One of the underlying molecular mechanisms of anoikis suppression can be explained by the loss of E-cadherin 33. Snail and Twist are required for this loss of E-cadherin, but whether Slug is capable of directly inducing anoikis suppression remains largely unclear. We demonstrated that Slug is a key molecule in the induction of anoikis suppression. In line with this finding, the elevated expression of *Slug* mRNA in lung cancer has been shown to be a poor prognostic factor for survival 6. Further, clinicopathological analysis of the Slug expression in lung cancer as a prognostic biomarker is warranted.

The recent studies on Slug knockdown in cancer cells identified the cancer-related target genes of slug; for example, Slug knockdown downregulated the expression of miR221, chemokine (C-X-C motif) receptor 4, and matrix metallopeptidase 9, and upregulated the expression of plakoglobin and ubiquitin-conjugating enzyme E2D3 34–37. In terms of the cellular phenotype, Slug enhanced cellular migration and invasion with acquisition of aggressive phenotype, in a manner similar to this study. Thus, accumulating bodies of evidence suggest that Slug regulates the expression levels of a wide variety of cancer-related target genes in addition to promoting EMT to cancer cells.

In conclusion, we demonstrated that Slug sensitizes lung cancer cells to TBAs via the suppression of βIII and βIVa-tubulin. Our findings provide novel insight into the biological function of Slug and suggest that Slug overexpression increases sensitivity to TBAs via the direct transcriptional regulation of βIII and βIVa-tubulin in lung cancer cells.

## Supporting Information

Table S1 Clinical characteristics of lung cancer patients treated with tubulin binding agents.

Table S2 The primers used for real-time RT-PCR.

Table S3 Summary of the β-tubulin isotype.

## References

[b1] Peinado H, Olmeda D, Cano A (2007). Snail, Zeb and bHLH factors in tumour progression: an alliance against the epithelial phenotype?. Nat. Rev. Cancer.

[b2] Hugo H, Ackland ML, Blick T, Lawrence MG, Clements JA, Williams ED (2007). Epithelial–mesenchymal and mesenchymal–epithelial transitions in carcinoma progression. J. Cell. Physiol.

[b3] Tsuji T, Ibaragi S, Hu GF (2009). Epithelial-mesenchymal transition and cell cooperativity in metastasis. Cancer Res.

[b4] Ouyang G, Wang Z, Fang X, Liu J, Yang CJ (2010). Molecular signaling of the epithelial to mesenchymal transition in generating and maintaining cancer stem cells. Cell. Mol. Life Sci.

[b5] Zeisberg M, Neilson EG (2009). Biomarkers for epithelial-mesenchymal transitions. J. Clin. Invest.

[b6] Shih JY, Tsai MF, Chang TH, Chang YL, Yuan A, Yu CJ (2005). Transcription repressor slug promotes carcinoma invasion and predicts outcome of patients with lung adenocarcinoma. Clin. Cancer Res.

[b7] Voulgari A, Pintzas A (2009). Epithelial-mesenchymal transition in cancer metastasis: mechanisms, markers and strategies to overcome drug resistance in the clinic. Biochim. Biophys. Acta.

[b8] Arumugam T, Ramachandran V, Fournier KF, Wang H, Marquis L, Abbruzzese JL (2009). Epithelial to mesenchymal transition contributes to drug resistance in pancreatic cancer. Cancer Res.

[b9] Yauch RL, Januario T, Eberhard DA, Cavet G, Zhu W, Fu L (2005). Epithelial versus mesenchymal phenotype determines in vitro sensitivity and predicts clinical activity of erlotinib in lung cancer patients. Clin. Cancer Res.

[b10] Wang Z, Li Y, Kong D, Banerjee S, Ahmad A, Azmi AS (2009). Acquisition of epithelial-mesenchymal transition phenotype of gemcitabine-resistant pancreatic cancer cells is linked with activation of the notch signaling pathway. Cancer Res.

[b11] Thomson S, Buck E, Petti F, Griffin G, Brown E, Ramnarine N (2005). Epithelial to mesenchymal transition is a determinant of sensitivity of non-small-cell lung carcinoma cell lines and xenografts to epidermal growth factor receptor inhibition. Cancer Res.

[b12] Fuchs BC, Fujii T, Dorfman JD, Goodwin JM, Zhu AX, Lanuti M (2008). Epithelial-to-mesenchymal transition and integrin-linked kinase mediate sensitivity to epidermal growth factor receptor inhibition in human hepatoma cells. Cancer Res.

[b13] Tanaka K, Arao T, Maegawa M, Matsumoto K, Kaneda H, Kudo K (2009). SRPX2 is overexpressed in gastric cancer and promotes cellular migration and adhesion. Int. J. Cancer.

[b14] Matsumoto K, Arao T, Tanaka K, Kaneda H, Kudo K, Fujita Y (2009). mTOR signal and hypoxia-inducible factor-1 alpha regulate CD133 expression in cancer cells. Cancer Res.

[b15] Tamura D, Arao T, Tanaka K, Kaneda H, Matsumoto K, Kudo K (2010). Bortezomib potentially inhibits cellular growth of vascular endothelial cells through suppression of G2/M transition. Cancer Sci.

[b16] Takeda M, Arao T, Yokote H, Komatsu T, Yanagihara K, Sasaki H (2007). AZD2171 shows potent antitumor activity against gastric cancer over-expressing fibroblast growth factor receptor 2/keratinocyte growth factor receptor. Clin. Cancer Res.

[b17] Kaneda H, Arao T, Tanaka K, Tamura D, Aomatsu K, Kudo K (2010). FOXQ1 is overexpressed in colorectal cancer and enhances tumorigenicity and tumor growth. Cancer Res.

[b18] Kudo K, Arao T, Tanaka K, Nagai T, Furuta K, Sakai K (2011). Antitumor activity of BIBF 1120, a triple angiokinase inhibitor, and use of VEGFR2+pTyr+ peripheral blood leukocytes as a pharmacodynamic biomarker. Clin. Cancer Res.

[b19] Ruifrok AC, Johnston DA (2001). Quantification of histochemical staining by color deconvolution. Anal. Quant. Cytol. Histol.

[b20] Kavallaris M, Kuo DY, Burkhart CA, Regl DL, Norris MD, Haber M (1997). Taxol-resistant epithelial ovarian tumors are associated with altered expression of specific beta-tubulin isotypes. J. Clin. Invest.

[b21] Gan PP, Kavallaris M (2008). Tubulin-targeted drug action: functional significance of class II and class IVb beta-tubulin in vinca alkaloid sensitivity. Cancer Res.

[b22] Gan PP, Pasquier E, Kavallaris M (2007). Class III beta-tubulin mediates sensitivity to chemotherapeutic drugs in non small cell lung cancer. Cancer Res.

[b23] Terry S, Ploussard G, Allory Y, Nicolaiew N, Boissière-Michot F, Maillé P (2009). Increased expression of class III beta-tubulin in castration-resistant human prostate cancer. Br. J. Cancer.

[b24] McCarroll JA, Gan PP, Liu M, Kavallaris M (2010). betaIII-tubulin is a multifunctional protein involved in drug sensitivity and tumorigenesis in non-small cell lung cancer. Cancer Res.

[b25] Mittal MK, Myers JN, Misra S, Bailey CK, Chaudhuri G (2008). In vivo binding to and functional repression of the VDR gene promoter by SLUG in human breast cells. Biochem. Biophys. Res. Commun.

[b26] Rho JK, Choi YJ, Lee JK, Ryoo BY, Na II, Yang SH (2009). Epithelial to mesenchymal transition derived from repeated exposure to gefitinib determines the sensitivity to EGFR inhibitors in A549, a non-small cell lung cancer cell line. Lung Cancer.

[b27] Kurrey NK, Jalgaonkar SP, Joglekar AV, Ghanate AD, Chaskar PD, Doiphode RY (2009). Snail and slug mediate radioresistance and chemoresistance by antagonizing p53-mediated apoptosis and acquiring a stem-like phenotype in ovarian cancer cells. Stem Cells.

[b28] Zhuo W, Wang Y, Zhuo X, Zhang Y, Ao X, Chen Z (2008). Knockdown of Snail, a novel zinc finger transcription factor, via RNA interference increases A549 cell sensitivity to cisplatin via JNK/mitochondrial pathway. Lung Cancer.

[b29] Dumontet C, Sikic BI (1999). Mechanisms of action of and resistance to antitubulin agents: microtubule dynamics, drug transport, and cell death. J. Clin. Oncol.

[b30] Leandro-García LJ, Leskelä S, Landa I, Montero-Conde C, López-Jiménez E, Letón R (2010). Tumoral and tissue-specific expression of the major human beta-tubulin isotypes. Cytoskeleton (Hoboken).

[b31] Seve P, Dumontet C (2008). Is class III beta-tubulin a predictive factor in patients receiving tubulin-binding agents?. Lancet Oncol.

[b32] Geiger TR, Peeper DS (2009). Metastasis mechanisms. Biochim. Biophys. Acta.

[b33] Onder TT, Gupta PB, Mani SA, Yang J, Lander ES, Weinberg RA (2008). Loss of E-cadherin promotes metastasis via multiple downstream transcriptional pathways. Cancer Res.

[b34] Lambertini E, Lolli A, Vezzali F, Penolazzi L, Gambari R, Piva R (2012). Correlation between Slug transcription factor and miR-221 in MDA-MB-231 breast cancer cells. BMC Cancer.

[b35] Bailey CK, Mittal MK, Misra S, Chaudhuri G (2012). High motility of triple-negative breast cancer cells is due to repression of plakoglobin gene by metastasis modulator protein SLUG. J. Biol. Chem.

[b36] Uygur B, Wu WS (2011). SLUG promotes prostate cancer cell migration and invasion via CXCR4/CXCL12 axis. Mol. Cancer.

[b37] Mittal MK, Singh K, Misra S, Chaudhuri G (2011). SLUG-induced elevation of D1 cyclin in breast cancer cells through the inhibition of its ubiquitination. J. Biol. Chem.

